# Targeting tumoral heterogeneity in lung cancer: a novel, CT-texture-guided targeted biopsy approach with exome sequencing

**DOI:** 10.1038/s41698-025-01148-5

**Published:** 2025-11-07

**Authors:** Alexander Hertel, Alexander Streuer, Steffen Diehl, Tobias Boch, Dominik Nörenberg, Anika Strittmatter, Frank G. Zöllner, Stefan O. Schoenberg, Wolf-Karsten Hofmann, Sonja Loges, Daniel Nowak, Matthias F. Froelich

**Affiliations:** 1 Department of Radiology and Nuclear Medicine, University Medical Center Mannheim, Heidelberg University, Mannheim, Germany; 2 Department of Hematology and Oncology, University Medical Center Mannheim, Heidelberg University, Mannheim, Germany; 3 Department of Personalized Oncology, University Hospital Mannheim, Medical Faculty Mannheim, University of Heidelberg, Mannheim, Germany; 4 Division of Personalized Medical Oncology (A420), German Cancer Research Center (DKFZ), German Center for Lung Research (DZL), Heidelberg, Germany; 5 DKFZ-Hector Cancer Institute at the University Medical Center Mannheim, University of Heidelberg, Mannheim, Germany; 6 Computer Assisted Clinical medicine, Medical Faculty Mannheim, Heidelberg University, Mannheim, Germany; 7 Mannheim Institute for Intelligent Systems in Medicine, Medical Faculty Mannheim, Heidelberg University, Mannheim, Germany

**Keywords:** Cancer imaging, Lung cancer, Tumour heterogeneity

## Abstract

Solid tumors like lung cancer show significant mutational heterogeneity. A biopsy captures only focal aspects, limiting conclusions about overall tumor biology. This prospective study correlated CT-based radiomics features with genomic profiles to optimize biopsy site selection. Lung cancer patients underwent CT imaging, radiomics analysis, targeted biopsies, and whole-exome sequencing. Twelve non-redundant features were extracted, with JointEntropy guiding biopsy targeting. In 7 of 12 patients, over 10% of mutations were exclusive to one biopsy. Clonal reconstruction showed heterogeneous profiles with over two subclonal processes in 67% of cases. Unsupervised clustering of radiomics features revealed two distinct groups separated by entropy features, of which the entropy-rich cluster was associated with STK11 mutations. Our study demonstrates that integrating radiomics with localized genomic analysis enhances the understanding of tumoral heterogeneity and may improve the targeting of advanced tumor regions for diagnostic sampling.

## Introduction

Lung cancer remains a formidable global health challenge, representing a significant burden on both individuals and healthcare systems^1,2^.

In lung cancer, particularly intra- and interlesional tumor heterogeneity significantly complicates the effectiveness of treatment, often leading to mixed responses and relapse^3,4^. Currently, staging and response assessment using radiologic methods largely relies on qualitative assessment and measurement of diameter on computed tomography (CT) or positron emission tomography (PET) in combination with CT. However, established diameter-based response assessment criteria such as RECIST 1.1 cannot comprehensively assess tumor heterogeneity and the underlying tumor biology^5^. Tissue-based diagnostic techniques, such as biopsies, provide the substrate for the mandatory histologic confirmation of the diagnosis but are limited to single localized tumor regions, which do not necessarily represent the most advanced tumor evolution. To this end, systemic liquid-based diagnostic techniques, such as analysis of circulating tumor DNA (ctDNA) and cell-free DNA (cfDNA) have shown improvements. However, their application remains challenging, and they do not provide information about the origin of mutated DNA^6,7^. For therapy planning, a thorough assessment of genetic variations, including driver mutations for targeted therapy, is pivotal^8–10^. Especially, but not limited to, a situation of relapse, it would be most favorable to obtain biopsies of the phylogenetically most advanced tumor regions to account for acquired mutations conferring resistance.

To address this problem, we utilize the emerging field of radiomics and its ability to visualize the textural characteristics of tissue. “Radiomics” refers to the extraction of quantitative data and parameters from radiological image datasets that go beyond the qualitative information detectable by the human eye^11^. Radiomics, and in particular texture features, have been shown to provide information about the biological characteristics of tumors and heterogeneity of lesions and can complement traditional biopsy- or serum-based approaches^12^.

In this study, we established a workflow for radiomics feature visualization and identification of potentially heterogeneous tumor areas in patients with lung cancer. We performed CT-guided multiple targeted biopsies of a set of areas with varying degrees of radiomics texture heterogeneity. Subsequently, we conducted exome sequencing to assess whether these radiomics-targeted biopsies could provide molecular evidence of intratumoral heterogeneity on a genetic level. Since the correlation between radiomics signatures and the identification of areas of heterogeneity in tumors such as glioblastomas has already been investigated, the next step is to establish a sufficient workflow that combines targeted biopsies with the creation of radiomics maps and the identification of potential areas of heterogeneity, which has not yet been established in clinical routine^13,14^. This innovative approach holds great promise for optimizing the accuracy of radiomics-guided biopsies and thus possibly optimizing personalized oncological therapy.

## Results

### Identification of non-redundant, visually representative texture features for biopsy targeting

**Fig. 1:**
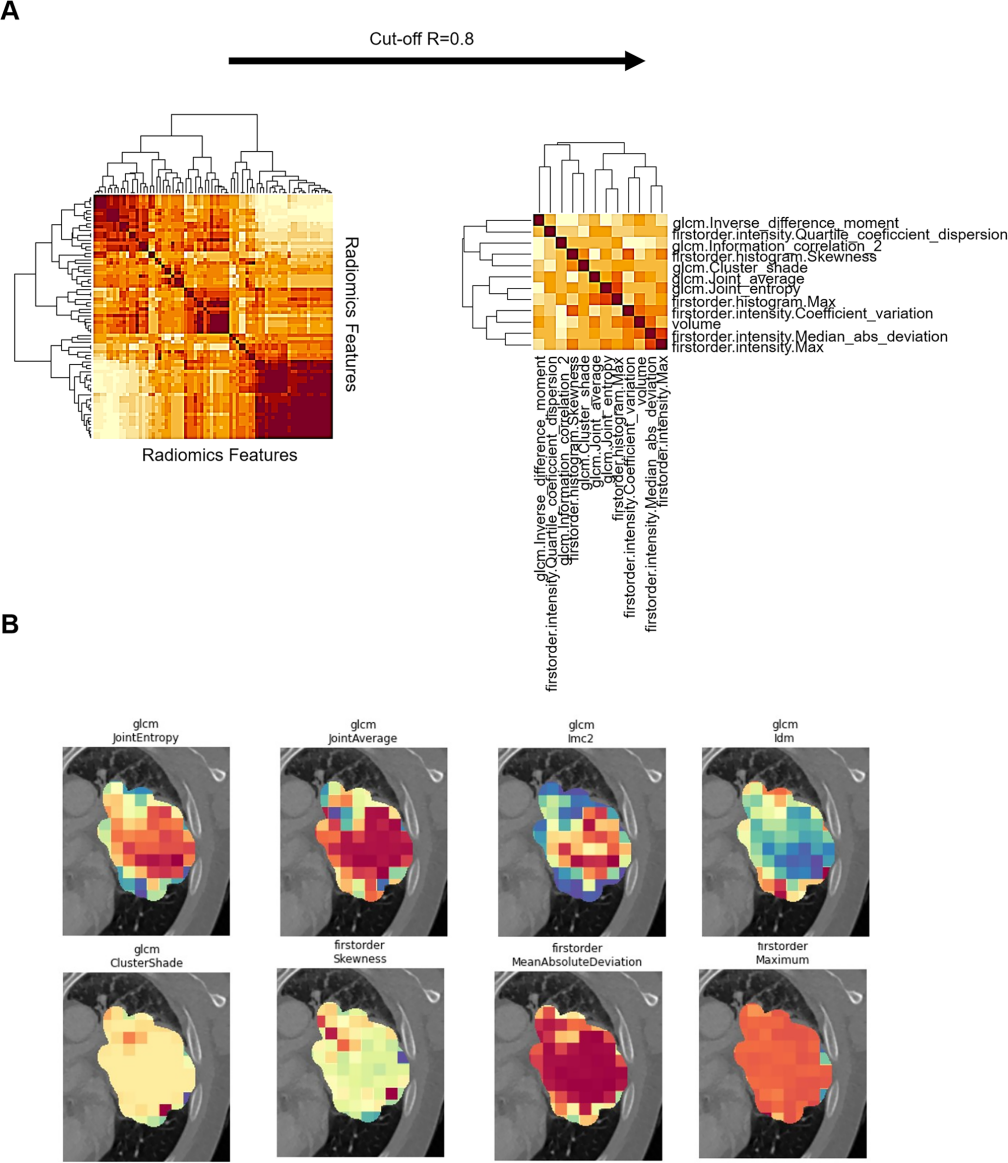
Radiomics Feature Selection. **A** Statistical feature selection with a cut-off value of *R*² = 0.6. **B** Visual feature selection with a representative collection of feature maps.

**Fig. 2:**
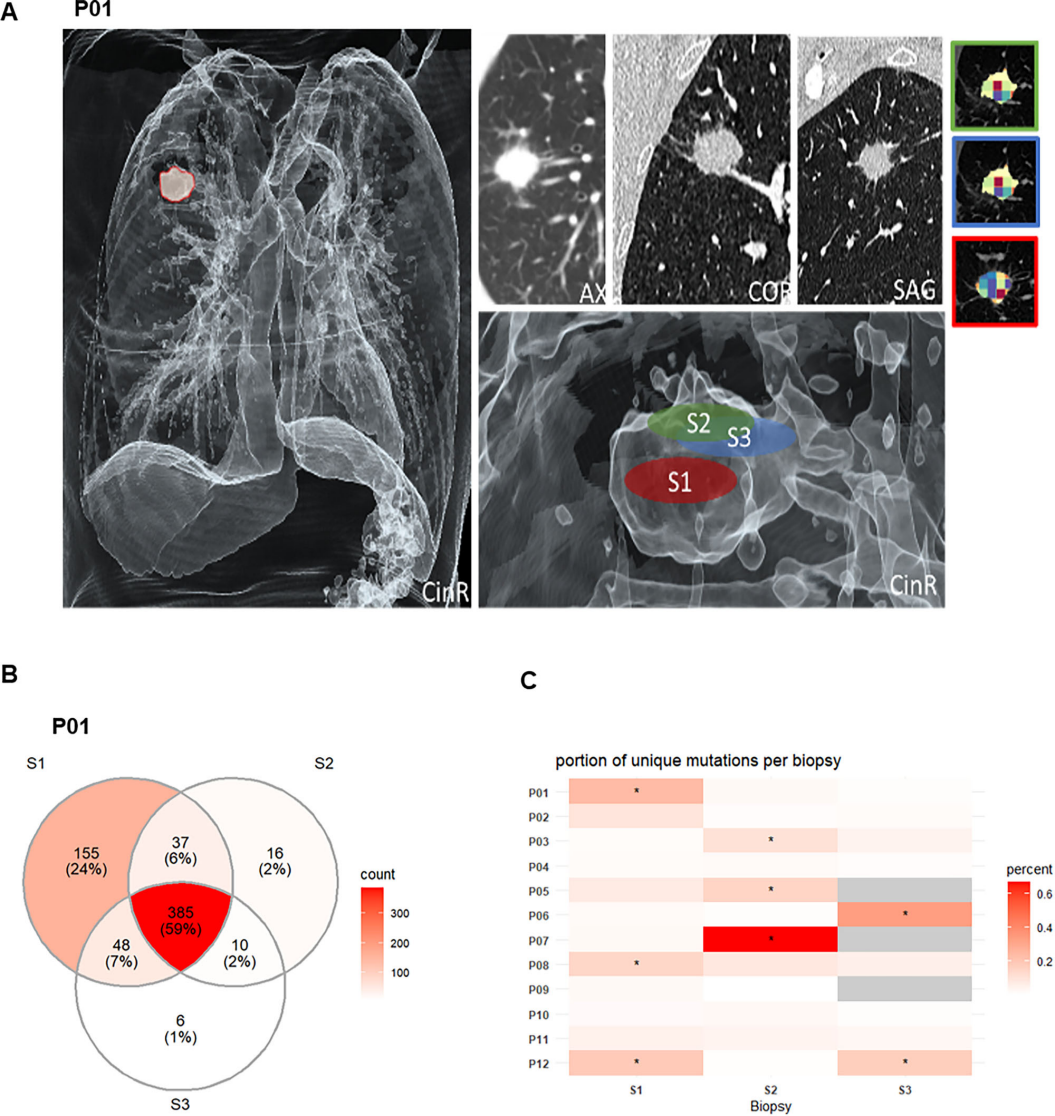
Qualitative molecular heterogeneity and VRT reconstructions of patient 01. **A** VRT reconstruction of the lung cancer of patient P01 (male, 68 yr, ADC), as well as the location of each taken sample and their corresponding visualization in the radiomics maps. **B** VENN diagram of detected common and exclusive mutations between the biopsies of P01. **C** Percentage of unique mutations detected per biopsy site.

From the extracted radiomics features, the feature reduction methodology resulted in the selection of 12 features (Fig. 1). These 12 features were utilized to create radiomics parameter maps of 23 representative lung tumor lesions (example patient Fig. 2). The selected features are listed in Supplemental Table 2. A structured assessment of two experienced radiologists in oncological imaging (M.F.F. and A.H.) under evaluation of the overall range of feature values, contrast within the lesion, visual textural diversity, and visual comparability resulted in the selection of *JointEntropy* for further assessment. Registration Accuracy was analyzed using a fiducial landmark-based approach. The mean fiducial registration error across all 12 patients was 1.52 mm (range: 0.5–3.8 mm), confirming adequate accuracy for retrospective virtual biopsy mapping. Detailed results of this analysis are provided in Supplemental Table 3.

### Significant molecular heterogeneity in intratumoral biopsies

To characterize intratumoral heterogeneity at the molecular level, two to three biopsies per patient were taken and exome sequenced. Figure 2A presents volume rendering technique (VRT) reconstructions of the tumor from Patient 1 (P01), along with the respective locations of the obtained samples and their corresponding visualization in the computed radiomics maps. On a genetic level, we first examined both common and exclusive mutations across different biopsies from the same patient. Here, significant differences were already evident at a qualitative level. For example, in patient P01, only 59% of all mutations were detected in all three biopsies. Moreover, 24% of mutations were exclusive to Biopsy 1 (Fig. 2B). Overall, in 7 out of 12 cases, more than 10% of exclusive mutations were observed in one of the biopsies (Fig. 2C).

**Fig. 3:**
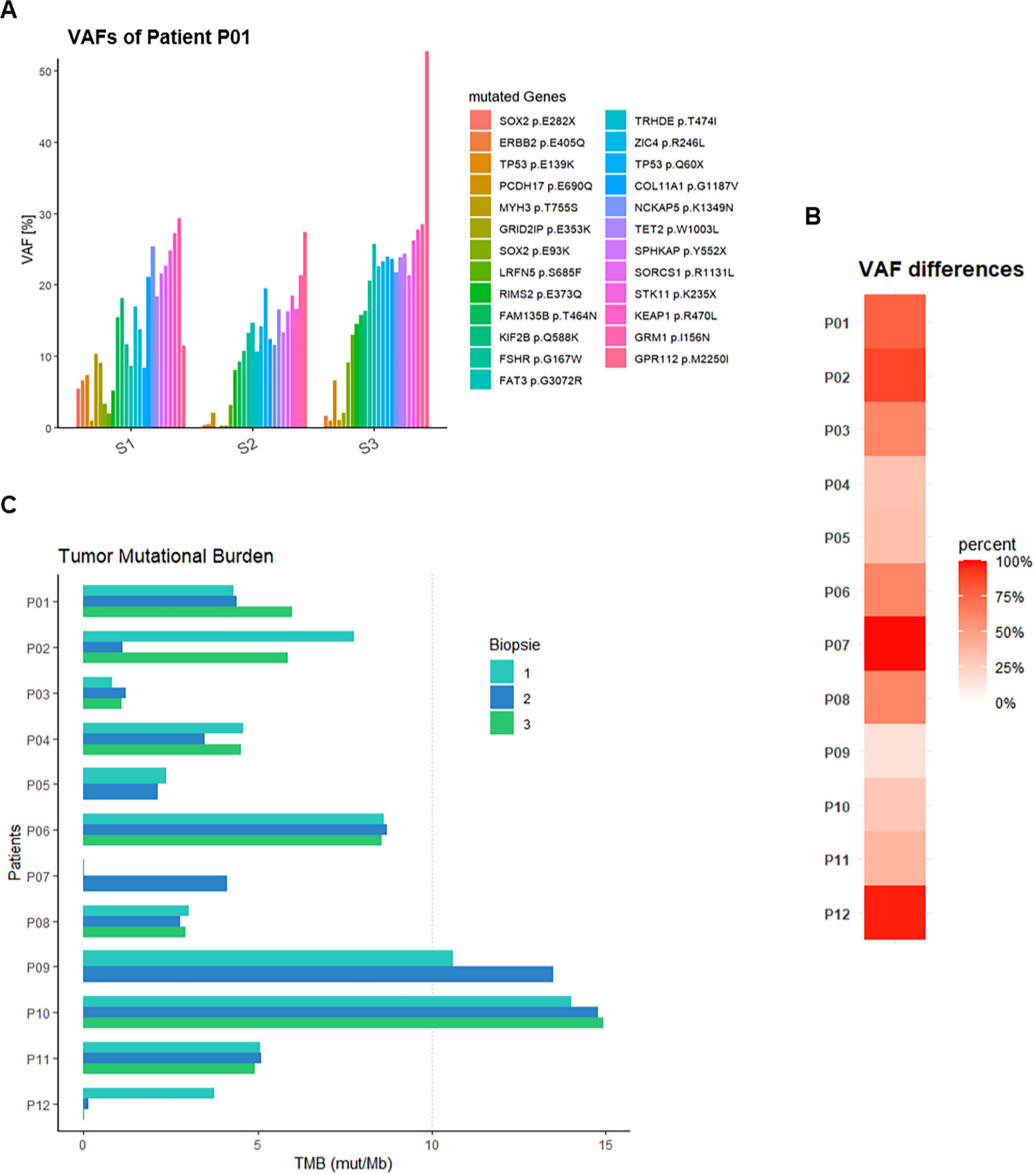
Quantitative molecular heterogeneity. **A** VAF of detected mutations. **B** Portion of VAF changes that differed by at least twofold between biopsies. **C** tumor mutational burden in mutations per megabase (mut/Mb).

In addition, significant differences were observed at a quantitative level in terms of variant allele frequency (VAF) between biopsies within the same patient. For instance, patient P01 exhibited a change in VAF between the different biopsies by a factor of 2 or more in 525 out of 682 (77%) mutations (Fig. 3A). Similarly, most of the other patients also showed significant differences. Overall, 8 out of 12 patients exhibited variations in VAF that differed by at least twofold between biopsies for the respective gene in more than 50% of all mutations (Fig. 3B). When calculating the tumor mutational burden (MTB) (number of mutations per megabase), no relevant differences were observed between biopsies in 9 out of 12 patients (Fig. 3C). However, it was noted that in three patients, one biopsy (P02, P07) or two biopsies (P15) exhibited substantially lower TMB values, containing only 12%, 1%, 4%, and 1% of the mutations detected in the corresponding paired samples of the same patient.

### Association of texture features and genetic variation within biopsy samples

**Fig. 4:**
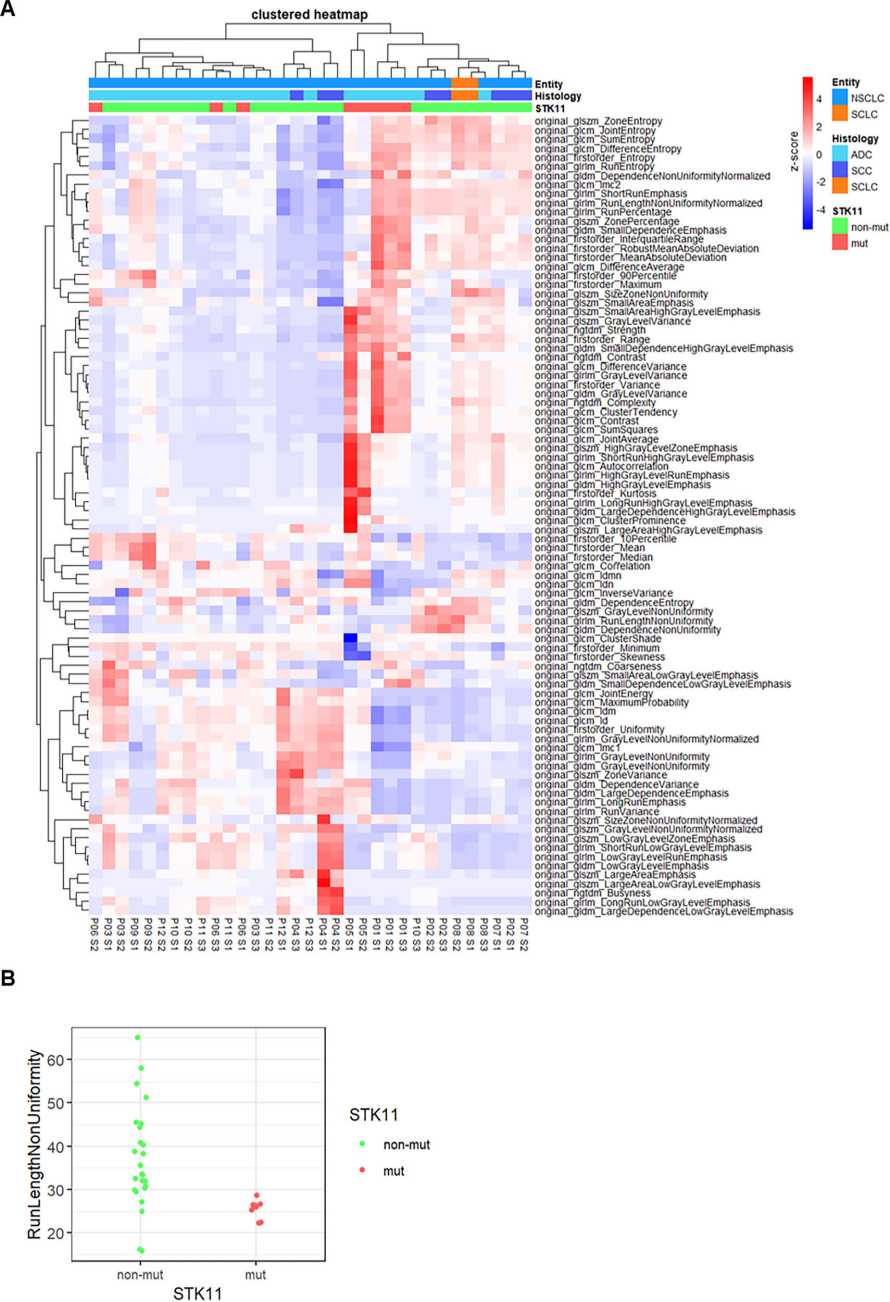
Cluster analysis. **A** Cluster analysis of extracted radiomics parameters and molecular genetic findings. **B** Dot plot of the radiomics feature RunLengthNonUniformity in non-mutated and mutated STK11 mutations (*p* = 0.0008).

To investigate the association between genomic and textural information, an unsupervised hierarchical clustering of texture features was performed, and a genetic annotation was added. Unsupervised hierarchical clustering was chosen as it enables unbiased grouping of biopsy samples based on radiomics texture features, facilitating the identification of natural patterns without pre-defined labels. This approach, combined with genetic annotation, allowed us to explore potential correlations between radiologic heterogeneity and genetic variation while providing interpretable visual representations in this exploratory study. This analysis resulted in the identification of *n* = 2 distinct clusters (Fig. 4A). These clusters were mainly separated by entropy features. Furthermore, samples from each patient tended to be clustered together. These two clusters and also distinct radiologic features were analyzed against various clinical parameters, including TMB, genetic heterogeneity assessed by exclusive mutations and VAF differences, subclonal patients status (>2 subclones) and patient outcomes evaluated in OS (median 1129 days) and PFS (median 624 days). No correlation was found. Nevertheless, we identified a subcluster of patients within the entropy-rich cluster defined by radiologic features that could be characterized by an STK11 mutation. Here, the feature RunLengthNonUniformity showed a strong elevation in non-mutated samples (Fig. 4B).

## Discussion

This work evaluates tumoral heterogeneity using a unique approach that connects CT-imaging texture of lung cancer lesions to the mutational landscape of multiple lesional regions.

The analysis reveals a significant degree of intratumoral heterogeneity, which is consistent with previous research^15^. Unlike earlier studies, this result was obtained through a texture-targeted, prospective approach rather than a post-mortem assessment^16^. In previous studies, the entire genomic profile in the form of cfDNA^12^ or single, local biopsies was correlated with radiological features^17^. Our analysis offers the opportunity for spatial resolution of genomic data, which has been correlated with quantitative radiological data. To our knowledge, this is the first analysis of its kind.

We observed that by performing unsupervised hierarchical clustering, the *n* = 33 biopsies formed *n* = 2 distinct clusters. In this process, samples from the same patient are often clustered together. Interestingly, entropy features best separate these two groups. Within the entropy-rich cluster, a subcluster was identified with a higher prevalence of STK11 mutations. The radiomics feature RunLengthNonUniformity was most prominently associated with STK11 unmutated samples, indicating a parameter for textural homogeneity within the CT cross-sectional imaging. STK11 (also known as LKB1) encodes a tumor suppressor involved in cellular energy homeostasis, polarity regulation, and cell adhesion. Loss-of-function mutations in STK11 are known to drive epithelial–mesenchymal transition (EMT), increase cellular motility, and reduce intercellular adhesion, ultimately resulting in greater spatial disorganization of tumor architecture^18^. This disordered microenvironment may translate into increased textural complexity on CT images, as captured by elevated entropy. Interestingly, RunLengthNonUniformity was relatively low in STK11-mutated lesions, possibly reflecting a more diffusely disorganized but texturally consistent tumor pattern. These imaging features are biologically possible, suggesting that STK11 alterations can promote both molecular and morphological heterogeneity in lung cancer^18,19^. Importantly, while driver mutations, such as KRAS and EGFR, were detected in several patients, no consistent correlation with specific radiomic features was observed.

Therefore, we demonstrate the feasibility of using imaging-based lesion heterogeneity assessment to improve biopsy targeting in lung cancer. This is an important topic as it has the potential to improve the sampling of tumor tissue to identify potential driver and resistance mutations, which is crucial for personalized therapy. It may also help to streamline the development of better personalized therapies. The study’s results can be interpreted on three levels. First, clinical routine cases demonstrate a relevant degree of tumoral heterogeneity, which is not taken into account in conventional biopsy approaches. Second, radiomics texture mappings can reveal regions within a lesion that may not be visible to the human eye on a standard CT scan and are potentially interesting. Third, this information can be utilized to improve the targeting of biopsies to obtain the most relevant tumor region in terms of driver and resistance mutations.

While our findings demonstrate the technical and clinical feasibility of radiomics-guided targeted biopsies, the relatively small cohort size (*n* = 12) inherently limits the generalizability and statistical robustness of specific associations. For instance, the observed link between STK11 mutations and entropy-rich radiomics subclusters must be interpreted with caution and regarded as exploratory. Due to the high dimensionality of radiomics data and the potential risk of overfitting, such associations require validation in larger, independent cohorts with appropriate correction for multiple testing and statistical learning methods. Future prospective studies should be designed to systematically investigate these genotype-phenotype links at scale. Given the relatively small sample size, we performed a post-hoc power analysis for the STK11-related finding. Assuming *α* = 0.05 and Cohen’s *d* = 1.0, ~34 samples are required to achieve 80% power. Our cohort of 33 biopsies is thus sufficient for detecting large effects, but smaller effects may remain underpowered. Therefore, these findings should be interpreted as exploratory.

To explore the broader applicability of this approach, we included both NSCLC and SCLC patients in this feasibility study, aiming to evaluate the utility of radiomics-guided targeted biopsy across a diverse real-world lung cancer cohort. However, we acknowledge that these subtypes differ significantly in their genetic profiles and imaging characteristics. Notably, NSCLC cases accounted for the majority of the cohort (11 out of 12 patients), and all exploratory radiogenomic associations, including the link between STK11 mutations and entropy-rich texture features, were derived from NSCLC cases. Future studies should be designed to include larger subtype-specific cohorts to allow stratified analysis and subtype-specific conclusions.

A key limitation of this study is the absence of an independent validation cohort, which restricts the generalizability of our findings. While we used a separate retrospective cohort of 23 patients for initial radiomics feature reduction, leading to the selection of JointEntropy, this cohort did not include detailed genomic data using whole-exome sequencing and thus cannot serve as a full external validation. Accordingly, our results should be regarded as preliminary and hypothesis-generating. Furthermore, as an exploratory feasibility study, no control group using standard or random biopsy targeting was included. Consequently, while the results suggest potential advantages of radiomics-guided biopsy, they do not allow for direct comparisons with conventional biopsy strategies. Future prospective studies with appropriate control arms are warranted to evaluate clinical benefit and diagnostic superiority. Additionally, since only radiologically heterogeneous regions were biopsied, the study does not allow conclusions about the genomic uniformity of radiologically homogeneous areas. This limits the ability to fully establish the specificity of radiomic features as predictors of genetic heterogeneity.

Also, identifying the most representative target lesions in the radiomics maps may be subjective and biopsy targeting can be challenging, particularly in the lung due to breathing and high risk of potential complications such as bleeding or pneumothorax. This leads to inaccuracies in the actual sampling, as the freedom of movement with the biopsy needle within the tumor is significantly restricted due to the high risk of complications. However, the topic was addressed using a dedicated fusion technique, and the biopsies were performed only by interventional radiologists with long-term experience. Additionally, radiomics features are subject to a certain degree of instability^20–22^. However, for targeting, semi-quantitative radiomics maps were employed, which may be less susceptible to quantitative instability. Also, the topic of instability may be addressed by the application of novel CT-imaging techniques such as photon counting CT^23,24^.

Quantitative radiomics markers are widely used in oncologic analyses, and the concept of intralesional heterogeneity is well-known from pathology assessment. However, quantitative imaging markers are not routinely used to address tumoral heterogeneity. Although deep learning mechanisms have partly outperformed radiomics analyses in terms of accuracy with regard to classification analyses, for example, of different tumor entities, these AI-based algorithms do not offer the possibility of definitive texture maps^25–27^. Until now, the assessment of intralesional heterogeneity in imaging has been mainly focused on magnetic resonance imaging (MRI) in certain cancer subtypes. This has been achieved through the application of perfusion or diffusion imaging techniques in multiple different malignant diseases^28–34^. However, CT is favored for its cost-efficiency and widespread availability, making it the only feasible approach for conducting CT-guided biopsies in lung cancer cases. Furthermore, CT may offer a higher degree of feature stability^35–37^. In summary, this work presents a novel, pioneering approach for texture-based biopsy targeting to address tumoral heterogeneity in lung cancer. This has the potential to improve personalized treatment and, therefore, the clinical outcome for patients.

## Methods

### Study design

**Fig. 5:**
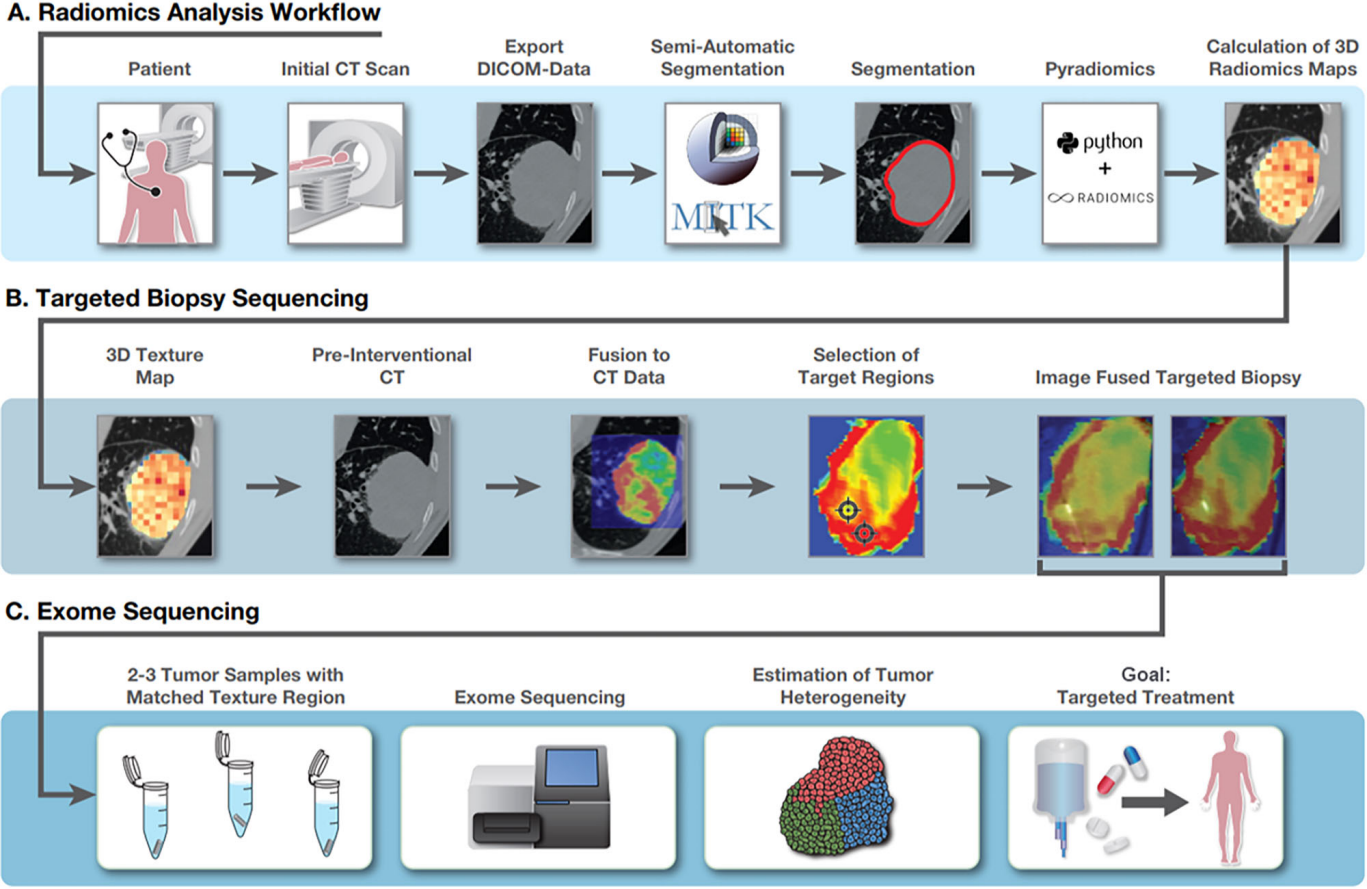
Schematic overview of the radiogenomics workflow for spatial biopsy targeting and tumor heterogeneity analysis. **A** Radiomics analysis workflow: patients underwent contrast-enhanced chest CT imaging. The DICOM data were exported, and semi-automated 3D tumor segmentation was performed using MITK. Radiomics features were extracted from the segmented tumor using the Pyradiomics package in Python. From these features, voxel-wise radiomics maps were generated, enabling spatial assessment of intratumoral heterogeneity. **B** Targeted biopsy sequencing: the most informative radiomics feature (JointEntropy) was selected and visualized as a 3D texture map. Prior to biopsy, an additional non-contrastenhanced CT was acquired. This pre-interventional scan was deformably registered to the contrast-enhanced planning CT. The fused dataset enabled retrospective localization of the biopsy needle relative to the radiomics map. Specific regions of interest—both entropy-high and entropy-low—were selected as biopsy targets. The final two images show axial slices with the biopsy needle overlaid on the radiomics map, illustrating the achieved spatial correlation (image fused targeted biopsy). **C** Exome sequencing: biopsy samples were matched to their corresponding radiomic subregion and processed for whole-exome sequencing. Tumor heterogeneity was assessed by analyzing subclonal architecture across samples. Ultimately, this integrative radiogenomic approach aims to support spatially informed and personalized treatment strategies.

In this prospective study, radiomic evaluations and visualization of radiomics parameters in the form of radiomics parameter maps were used to identify imaging-based tumor heterogeneity that may allow improvement in the precision of image-guided biopsy procedures. 12 patients with confirmed malignant neoplasia of the lung who have received cross-sectional imaging as part of their clinical follow-up were included. The inclusion criterion was the presence of non-small-cell-lung-cancer (NSCLC) or small-cell-lung-cancer (SCLC) larger than 2 cm in maximum diameter. 2 cm was selected as the threshold to ensure that the tumor was large enough to allow the identification of heterogeneity areas in the radiomics maps and targeted puncturing. An evaluation of existing image data sets as well as image data sets obtained during routine care, was performed. After radiomics analysis and mapping, a biopsy of the identified areas of heterogeneity was conducted to perform molecular genetic analysis. This prospective research project was approved by the Institutional Review Board (2021-639-AF 5) of the Ethics Committee II (University Hospital Mannheim) and the study was conducted in accordance with the Declaration of Helsinki. All patients were explicitly informed verbally and in written form about the study protocol and the associated measures in the context of CT-guided biopsy and gave their consent. Progression-free survival (PFS) and overall survival (OS) were assessed retrospectively from clinical records. PFS was defined as the time from biopsy to radiologically or clinically confirmed disease progression or death, and OS as the time from biopsy to death from any cause. The study design is shown in detail in Fig. 5. The patient collective is summarized in Table 1.

**Table 1:** Patient characteristics

Pat_ID	Age	Sex	entity	TNM	Biopsy	Pathogenic mutations	Previous therapy
P01	68	m	ADC, NSCLC	cT1cNxcM1b	1	TP53 p.Q192*, TP53 p.E139K, STK11 p.K235*, KEAP1 p.R470L, CUL3 p.L526fs	None
					2	TP53 p.Q192*, STK11 p.K235*, KEAP1 p.R470L	
					3	TP53 p.Q192*, STK11 p.K235*, KEAP1 p.R470L	
P02	59	f	SCC, NSCLC	cT2cN3cM0	1	KRAS p.G12V, TP53 p.G105V, SMAD4 p.G352A,	None
					2	KRAS p.G12V, TP53 p.G105V, SMAD4 p.G352A,	
					3	KRAS p.G12V, TP53 p.G105V, SMAD4 p.G352A	
P03	51	f	ADC, NSCLC	cT4cN3M1c	1	EGFR del Exon 19, TP53 p.H47Y	Osimertinib
					2	EGFR del Exon 19, TP53 p.H47Y	
					3	EGFR del Exon 19, TP53 p.H47Y	
P04	61	m	SCC, NSCLC	cT3cN1cM1a	1	TP53 p.P250fs, CDKN2A p.G6fs	None
					2	TP53 p.P250fs, CDKN2A p.G6fs	
					3	TP53 p.P250fs, CDKN2A p.G6fs	
P05	67	m	ADC, NSCLC	pT1cN0M0	1	KRAS p.G12V, STK11 p.Y60*, CDC42 p.K144M	None
					2	KRAS p.G12V, STK11 p.Y60*	
P06	66	m	ADC, NSCLC	cT3cN1cM1b	1	KRAS p.G12V, STK11 p.G279fs	None
					2	KRAS p.G12V, STK11 p.G279fs	
					3	KRAS p.G12V, STK11 p.G279fs	
P07	82	m	SCC, NSCLC	cT4cN2cM1	1	No sig. mutations	None
					2	TGFBR2 p.R497X	
P08	69	m	SCLC	cT4cN0cM0	1	DDHD2 p.T186M	None
					2	DDHD2 p.T186M,	
					3	DDHD2 p.T186M, COL1A1 p.G1169S	
P09	65	f	ADC, NSCLC	cT3cN3pM1c	1	KRAS p.G12C, GNAS p.R201L	Carbo + Pem + Pembro, Sotorasib
					2	KRAS p.G12C, GNAS p.R201L	
P10	64	m	ADC, NSCLC	pT3pN0cM0	1	TP53 p.F270V	None
					2	TP53 p.F270V, ARID2 p.Q1637*	
					3	TP53 p.F270V, ARID2 p.Q1637*	
P11	56	m	ADC, NSCLC	cT4N3M1c	1	TP53 p.Y163*, KIT p.W557G	None
					2	TP53 p.Y163*, MAP2K1 p.E203K	
					3	TP53 p.Y163*, KIT p.W557G	
P12	66	m	ADC, NSCLC	pT4cN0cM0	1	KRAS p.Q61H, STK11 p.D194Y	None
					2	ARID4A p.E426*	
					3	No sig. mutations	

Patient characteristics include age, sex, tumor entity, TNM classification, biopsies, pathogenic mutations, and previous therapies.

*ADC* adenocarcinoma, *NSCLC* non-small cell lung cancer, *SCLC* small cell lung cancer, *m* male, *f* female.

* indicates Stop Codon.

### CT-Imaging

Patients received a CT scan of the lung as part of their routine clinical care. CT images were acquired with either a Siemens SOMATOM Definition AS, SOMATOM Definition Flash, or a SOMATOM Definition 64 (Siemens Healthcare GmbH, Erlangen, Germany) using a contrast-enhanced routine CT. Axial images of all scans were reconstructed with a slice thickness of 1 mm (increment 1 mm) using a soft tissue kernel (I31f) as well as a lung tissue kernel (I70f) as part of routine imaging. While no explicit feature harmonization or stability analysis was performed, the use of voxel-wise radiomics maps for intra-individual biopsy targeting, rather than cross-patient comparisons, minimizes the expected impact of scanner-related variability. The contrast agent used was Imeron 300 (Iomeprol, Bracco Imaging S.p.A., Milan, Italy) at a dose adjusted according to CT protocol and patient weight and involved the application of 80–100 ml of Imeron.

### CT image analysis

Image Data was anonymized and exported from the hospital’s internal PACS system with the image viewer software Aycan Workstation Pro® (Version 3.14.006, Aycan Medical Systems, LLC, NY 14607, USA) and stored in the DICOM (Digital Imaging and Communication in Medicine) file format. Segmentation of the lung tumors was performed semi-automatically by a clinical radiologist with three years of segmentation experience. Subsequently, the segmentation and image data were converted to the Neuroimaging Informatics Technology Initiative (NIFTI) file format for further processing using a dedicated segmentation tool (MITK Workbench, version 2021.10).

### Texture analysis

Radiomics features of the prepared segmentations were extracted using a Python package for imaging biomarkers (Pyradiomics, version 3.0.1^38^) integrated into a purpose-built Docker container (Docker Desktop; version 4.3.1, Docker, Inc., Palo Alto, CA, USA). For feature extraction, a CT series with 1 mm slice thickness and I37f soft tissue kernel was used. For each segmentation, standard radiomics features, including shape features, first-order features, as well as second-order features (>1000 features in total) were extracted, namely Firstorder, grayscale co-occurrence matrix (glcm), grayscale dependence matrix (gldm), grayscale size zone matrix (glszm), grayscale run length matrix (glrlm), and grayscale neighborhood difference matrix (ngtdm). Extraction was performed with voxel normalization, resampling to 2 × 2 × 2 mm, and rebinning with a fixed bin width of 25 HU according to pyRadiomics standards. The Chebyshev distance is 1. The used Radiomics Feature Extraction Workflow complies with the Image Biomarker Standardisation Initiative (IBSI)^39^. The features were then revisualized as 2D and 3D maps using a customized Python script based on Pyradiomics (deposited in a specially created Docker container). Radiomics parameter maps were calculated by mapping the extracted radiomics parameters to the spatial dimensions of the segmented tumor volumes.

For each voxel within the segmentation, the respective radiomics feature value was calculated based on the image intensities and relationships within its defined neighborhood (e.g., grayscale co-occurrence matrices for texture features). This voxel-based approach enabled the creation of parameter maps, where each voxel’s value corresponds to a specific radiomics feature.

These maps were then visualized with a pixel size of 5 mm using a color scale, with each color representing a specific range of feature values. Since the calculated radiomics maps were viewed individually for each patient to plan targeted puncture and no comparison was made between individual patients with regard to radiomics stability, no grayscale harmonization or stability tests with phantoms were performed.

### Feature selection

To identify potentially heterogeneous tumor areas as targets for CT-guided biopsies, radiomics feature selection was performed on the patient cohort as well as on a separate retrospective cohort of 23 patients with lung cancer (not included in the prospective analysis; *n* = 23, male: *n* = 16, female: *n* = 7, NSCLC: *n* = 17, SCLC: *n* = 6). The feature selection process in this study was not intended for classification or prediction tasks. Instead, it served to identify non-redundant and visually interpretable radiomics features that could highlight intratumoral heterogeneity and thereby guide CT-biopsy targeting. No specific clinical or molecular outcome was used as a target variable. The selection approach was thus unsupervised and exploratory in nature. The available image datasets were segmented semi-automatically, and radiomics parameters were extracted using Pyradiomics. Initially, radiomics features were extracted from the entire tumor ROI to perform statistical feature selection. Subsequently, the selected features were recalculated in a voxel-wise manner to generate spatial radiomics maps for visual assessment and virtual biopsy planning. Unsupervised hierarchical clustering was performed in *R*-Statistics to analyze the structure of the data and identify dependencies between the radiomics features. A distance matrix was first calculated based on the Pearson correlation, which quantified the similarity between individual radiomics features. The resulting hierarchical dendrogram provided an intuitive visualization of feature similarities, enabling the identification of highly correlated features that potentially carry redundant information. Based on the clustering analysis, features with a high degree of correlation (correlation coefficient *R* > 0.8) were removed to filter out parameters with high collinearity while retaining features with maximal relevance for subsequent analysis. The R-packages used for hierarchical clustering are detailed in Supplemental Table 1. This resulted in the selection of 12 non-redundant features used for visual mapping and targeted biopsy planning. As this study did not involve predictive modeling, no cross-validation or supervised learning was applied. Supplemental Table 2 shows the statistically selected features. In addition to statistical feature selection, a visual selection of the computed maps was performed. For visual feature selection, two radiologists with over 5 years of experience evaluated every calculated radiomics map for the overall range of feature values within the tumor, contrast and internal variability of the feature within the lesion, as well as visual comparability. Based on this visual evaluation, the feature JointEntropy was selected for radiomics map generation and further analysis due to its consistent expression of intralesional textural variation.

### CT-guided biopsy

The previously calculated radiomics maps of the selected parameters were exported in DICOM format and subsequently reviewed by the interventional radiologist as preparation immediately before CT-guided biopsy to identify and target the potentially heterogeneous areas of the tumor. The segmentations and evaluations were performed by a diagnostic radiologist with more than 5 years of experience in thoracic imaging and then discussed in detail with the interventional radiologist, who was responsible for performing the targeted biopsy. Biopsy target regions were selected based on the voxel-wise JointEntropy maps, which revealed spatial variations in intratumoral heterogeneity. Radiologists identified areas of high and low entropy as potential targets, aiming to capture both heterogeneous and homogeneous tumor subregions. Practical considerations such as needle trajectory, accessibility, and procedural safety were incorporated into the final selection of biopsy targets. The biopsy was performed under standard conditions without application of a contrast agent according to the hospital’s SOP, with the additional collection of samples from previously identified distinct areas of the tumor. To maintain the clinical realism of our study and ensure translatability and reproducibility, all biopsies were performed using the same breath-hold instructions and procedural standards as in routine diagnostic lung biopsies at our institution. All biopsies were performed in a Siemens Somatom Flash CT scanner.

### Virtual biopsy

After the successful biopsy, image fusion of the original planning CT image dataset and the biopsy image dataset was performed to correlate the exact location of the tip of the biopsy needle inserted during each sampling with the original CT images. In the next step, an image fusion was performed. We performed a registration of the CT images and their radiomics maps on the biopsy CT images. In some cases, due to needle placement, patients were lateral during the biopsy, whereas they were supine during the preliminary examination. We first performed rigid pre-registration of the CT images and their radiomics maps to correct the large rotations and translations, as the capture range of registration methods is not sufficient to correct such large rotations. The rotation was determined using bounding boxes that were automatically created around the CT and biopsy CT images^40^. Additionally, ITK-Snap was used to estimate corresponding slices in the images.

The pre-registered CT images were then registered to the biopsy CT images using a SimpleElastix multistage algorithm that combines rigid, affine, and deformable transformations^41^. The transformations calculated by the rigid pre-registration and SimpleElastix were then applied to the radiomics maps. To take potential changes of the biopsied region from the initially targeted regions into account (*R* = 0.003), the actual biopsied regions were utilized as ground truth, as they represent the topographic correspondence of the regions for which the exome sequencing was performed. To assess the accuracy of the deformable registration between the pre-interventional and planning CT scans, a fiducial landmark-based analysis was performed using 3D Slicer (version 5.6.2, www.slicer.org). Distinct anatomical landmarks, such as bronchial bifurcations, bone structures and vascular branching points were manually identified in both image datasets. For each patient, 3–4 corresponding fiducial points were placed in both the fixed (planning CT) and the moving (pre-interventional CT) images. After deformable registration with SimpleElastix, the Euclidean distance between corresponding landmarks was calculated in 3D space. The root mean square (RMS) of these point-wise distances was defined as the fiducial registration error for that patient.

### Molecular analysis

To measure intratumoral heterogeneity, primary samples from *n* = 12 patients were analyzed. From each patient, either two or three CT-guided biopsies were subjected to whole-exome sequencing. CD3+ positive cells from peripheral blood samples served as a germline control.

For library preparation, the Illumina DNA Flex for Enrichment Kit with probes from the IDT xGen Exome Research panel was used with 150 ng DNA input. Sequencing was performed on an Illumina NovaSeq 6000 platform in the 150 bp paired-end configuration. On average, a coverage of 166 (range 137–206) was achieved.

The sequencing data were mapped with bwa v0.7.5 and then deduplicated and recalibrated using Picard v1.100 and the GATK toolkit v3.8^42–44^. For quality control, the tools Fastqc v0.11.9 and Qualimap v2.2.1 were used^45,46^.

Somatic mutations were identified using Mutect2^47^. Cytogenetic changes were analyzed using FACETS^48^. Clonal reconstruction analysis was performed with PyClone-VI^49^. Phylogenetic trees were calculated using the R packages ape and phangorn^49^. The relevance of each variant was determined according to the Standards for the Classification of Pathogenicity of Somatic Variants in Cancer (Oncogenicity)^50^.

### Combined statistical analysis

The statistical analysis was performed with R Statistics using RStudio (Version 4.3.2, RStudio Team (2023). RStudio: Integrated Development Environment for R. RStudio, PBC, Boston, MA. URL: http://www.rstudio.com/). Genetic variation was visualized as heatmaps. Correlations of radiomics target variables and tumor mutational burden (TMB) were visualized as line plots. Overlap of mutations was visualized as Venn diagrams. For categorical variables, Fisher’s exact test was applied, while continuous variables were analyzed using *t*-tests or Wilcoxon rank-sum tests for two-group comparisons and ANOVA for comparisons involving more than two groups. Correlations of survival with clinical and molecular data were assessed using Kaplan–Meier estimates with log-rank tests.

## Supplementary information

Supplementary information

## Data Availability

The datasets and code used and/or analyzed during the current study are available from the corresponding author on reasonable request.
