# Supplementary material for: Targeting tumoral heterogeneity in lung cancer: a novel, CT-texture-guided targeted biopsy approach with exome sequencing
Source: NPJ Precis Oncol. 2025 Nov 7;9:342. doi: 10.1038/s41698-025-01148-5 (PMC12594912; doi:10.1038/s41698-025-01148-5)
Supplement: Supplementary file 1 — Supplementary information [file 41698_2025_1148_MOESM1_ESM.pdf]

Supplemental Table 1: R statistics packages used for the statistical analysis.

R statistics package	Version	Comment
“tableone”	0.13.0	Construction of a patient baseline characteristics table
“readxl”	1.3.1.9000	Improved compatibility for Excel files and R
“corrplot”	0.92	A visual exploration tool for correlation matrices that supports automatic reordering of variables to detect hidden patterns between variables.
“caret”	6.0-82	Includes functions to provide model training for complex regression and classification problems as well as creating predictive models
“pheatmap”	1.0.12	For Creation of radiomics heatmaps
“ComplexHeatmap”	2.6.2	Visualization of Heatmaps

Supplemental Table 2: The resulting radiomics features from the statistical feature selection and their feature class.

No.	Radiomics Feature	Feature Class
1	glcm.Inverse_difference_moment	GLCM
2	firstorder.intensity.Quartile_coefficient_dispersion	First-order
3	glcm.Information_correlation_2	GLCM
4	firstorder.histogram.Skewness	First-order
5	glcm.Cluster_shade	GLCM
6	glcm.Joint_average	GLCM
7	glcm.Joint_entropy	GLCM
8	firstorder.histogram.Max	First-order
9	firstorder.intensity.Coefficient_variation	First-order
10	volume	Shape
11	firstorder.intensity.Median_abs_deviation	First-order
12	firstorder.intensity.Max	First-order

Supplemental Table 3: Results of the registration accuracy analysis. (RMS=root mean square; TRE=target registration error; SD=standard deviation).

ID	RMS	TRE mean	SD	TRE max
Patient 1	0.826796	0.96	0.79	1.87
Patient 2	0.824606	1.8	0.54	2.4
Patient 3	1.15347	2	0.14	2.15
Patient 4	1.71554	3.3	0.08	3.39
Patient 5	1.83038	2.5	1.16	3.32
Patient 6	1.28229	1.86	0.32	2.22
Patient 7	2.48897	3.56	0.42	4.12
Patient 8	0.994406	1.93	0.95	2.87
Patient 9	1.73501	3.79	0.85	4.37
Patient 10	0.884845	1.7	0.14	1.85
Patient 11	2.05129	2.26	0.15	2.43
Patient 12	2.51158	3.1	0.42	3.58
